# Novel Compound Heterozygous *CBS* Mutations Cause Homocystinuria in a Han Chinese Family

**DOI:** 10.1038/srep17947

**Published:** 2015-12-15

**Authors:** Bo Gong, Liping Liu, Zhiwei Li, Zimeng Ye, Ying Xiao, Guangqun Zeng, Yi Shi, Yumeng Wang, Xiaoyun Feng, Xiulan Li, Fang Hao, Xiaoqi Liu, Chao Qu, Yuanfeng Li, Guoying Mu, Zhenglin Yang

**Affiliations:** 1 Sichuan Provincial Key Laboratory for Disease Gene Study, Sichuan Academy of Medical Sciences & Sichuan Provincial People’s Hospital, School of Medicine, University of Electronic Science and Technology of China, Chengdu, China; 2 Department of Ophthalmology, Shandong Provincial Hospital Affiliated to Shandong University, Jinan, Shandong, China; 3 Department of Ophthalmology, School of Medicine, Sichuan Academy of Medical Sciences & Sichuan Provincial People’s Hospital, University of Electronic Science and Technology of China, Chengdu, China

## Abstract

The *cystathionine β-synthase* (*CBS*) gene has been shown to be related to homocystinuria. This study was aimed to detect the mutations in *CBS* in a Han Chinese family with homocystinuria. A four-generation family from Shandong Province of China was recruited in this study. All available members of the family underwent comprehensive medical examinations. Genomic DNA was collected from peripheral blood of all the participants. The coding sequence of *CBS* was amplified by polymerase chain reaction (PCR), followed by direct DNA sequencing. Among all the family members, three affected individuals showed typical clinical features of homocystinuria. Two novel compound heterozygous mutations in the *CBS* gene, c.407T > C (p. L136P) and c.473C > T (p.A158V), were identified by sequencing analysis in this family. Both of the two missense mutations were detected in the three patients. Other available normal individuals, including the patients’ parents, grand parents, her younger sister and brother in this family either carried one of the two mutations, or none. In addition, the two mutations were not found in 600 ethnically matched normal controls. This study provides a mutation spectrum of *CBS* resulting in homocystinuriain a Chinese population, which may shed light on the molecular pathogenesis and clinical diagnosis of *CBS*-associated homocystinuria.

Homocystinuria, most commonly caused by cystathionine β-synthase (*CBS*) deficiency, is an autosomal recessive disorder of sulfur amino acid metabolism. *CBS* protein is a pyridoxal 5′ phosphate dependent enzyme and catalyzes the condensation of homocysteine with homocysteine and serine to form cystathionine^1^. Biochemically, this disorder is characterized by elevated plasma concentrations of homocysteine and methionine, increased excretion of homocysteine in urine and decreased levels of cystathionine and cysteine in body fluids^2^. Patients with homocystinuria often display different symptoms,including ocular anomalies (severe myopia and ectopialentis), skeletal deformities (osteoporosis, scoliosis and Marfanoid habitus), vascular thrombosis and ischemia, disorder of central nervous system (mental retardation, convulsions and psychiatric disturbances) and other manifestations^3^.

It has been reported that homocystinuria due to *CBS* deficiency is caused by mutations in the *CBS* gene^4^. *CBS* mutations could lead to the disruption of enzyme activity which consequently results in increased levels of homocysteine, a potentially toxic amino acid responsible for patients with homocystinuria. So far, more than 150 mutations in the *CBS* gene have been identified (http://cbs.lf1.cuni.cz/mutations.php) in different ethnic population^5^,^6^,^7^,^8^,^9^,^10^,^11^,^12^. Although many mutations have been described in the *CBS* gene from homocystinuric patients and this disease has been well characterized in other populations, the range of clinical presentations and spectrum of *CBS* mutations in Han Chinese patients remained largely uninvestigated.

In this study, we characterized the clinical manifestations and investigated the molecular basis of a Han Chinese family with homocystinuria, to expand the *CBS* mutation spectrum of the homocystinuric patients from China.

## Materials and Methods

### Subjects

**Figure 1:**
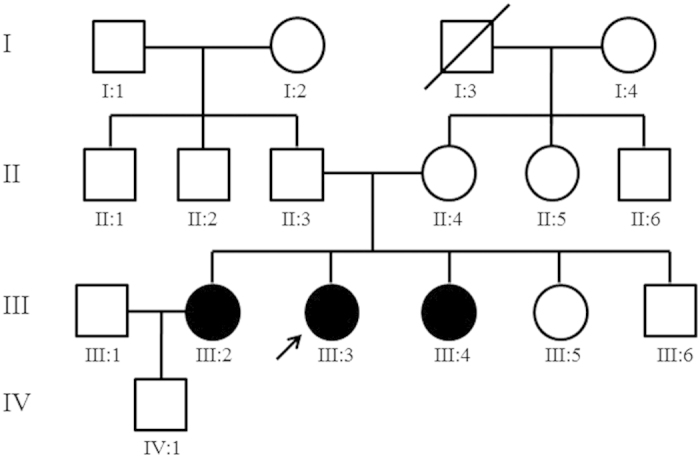
Pedigree of the family with homocystinuria. Solid symbols indicated affected individuals, and open symbols indicate unaffected individuals. Arrow indicates the proband.

This family with homocystinuria, including 16 members, was recruited from Shandong Provincial Hospital Affiliated to Shandong University (Fig. 1). Three of the family members were diagnosed as homocystinuria by biochemical profiles (grossly increased serum homocysteine and methionine) and by critical complications, such as dislocated lens, mental retardation and skeletal deformities. Their clinical information is summarized in Table 1.This study was conducted in accordance with the tenets of the Declaration of Helsinki and approved by the Institutional Review Boards of Hospital of University of Electronic Science and Technology of China & Sichuan Provincial People’s Hospital, and Shandong Provincial Hospital Affiliated to Shandong University. Written informed consents were obtained from the family prior to the study.Unrelated healthy control subjects were recruited from the Hospital of University of Electronic Science and Technology of China and Sichuan Provincial People’s Hospital. These controls are all Han Chinese, and subjects were excluded from the study if they had any of the symptoms, including ocular anomalies, skeletal deformities, vascular thrombosis and disorder of central nervous system.

### DNA Extraction

All genomic DNA was extracted from peripheral blood using a blood DNA extraction kit (QIAamp DNA Blood Midi Kit; Qiagen, Germany) according to the manufacturer’s protocol. DNA samples were stored at −20 °C until used. DNA integrity was evaluated by 1% agarose gel electrophoresis.

### Mutation screening

The method for mutation screening was performed as described previously^13^. Besides the variants in *CBS,* mutations in the *MTHFR* gene also have been reported to cause homocystinuria^14^,^15^. Therefore, the coding sequences of *CBS* (NM_000071.2) and *MTHFR*(NM_005957.4) were amplified by polymerase chain reaction (PCR) using a MyCycler thermo cycler (Bio-Rad, Hercules, CA). We did not detect any mutation in the *MTHFR* gene in this family (data not shown), thus it was excluded to cause homocystinuria in this study. Sequencing primers from flanking sequence of each exon of the *CBS* gene were designed by using the Primer 5.0 (Table 2). Amplification reaction was performed by the PCR reaction (10 μL final volume) containing 50 ng of genomic DNA, 1 μL of each primer (10 pmol/μL), 1 μL of 10 buffer (Takara Bio Inc., Shiga, Japan), 0.8 μL of deoxyribonucleotide triphosphates (2 mmol/L; Takara Bio Inc.), 0.4 μL MgCl_2_ (2.5 mmol/L; Takara Bio Inc.), and 0.1 μL of ExTaq polymerase (5 U/μL; Takara Bio Inc.). Amplified PCR products were purified with spin columns (QIAquick, Qiagen, Valencia, CA) and sequenced directly (BigDye Terminators Sequencing Kit; Applied Biosytems) in both directions with an automated genetic analysis system (ABI 3130 Genetic Analyzer, CA, USA).

Multiple sequence alignment of the human *CBS* protein was performed along with other *CBS* protein across different species, to check for the conservation of the residues. The possible damaging effects of the 2 mutations on the structure and function of *CBS* were predicted using SIFT (http://sift.jcvi.org) and PolyPhen-2 (http://genetics.bwh.harvard.edu/pph2/).

## Results

### Clinical findings

A four-generation family from Shandong Province of China was recruited in this study (Fig. 1). There are three affected individuals (III:2, III:3 and III:4), who showed typical clinical symptoms of homocystinuria among all the family members. The proband (III:3), as well as her two affected sisters(III:2 and III:4) exhibited similar clinical features, such as various reduced visual acuities with a bilateral lens dislocation, myopia, glaucoma, skeletal deformities and mental retardation(Table 1). All the patients also have elevated plasma homocysteine and methionine levels, compared to all the normal individuals of this family (±13.6 μmol/L for homocysteine and ±24.7 μmol/L for methionine, respectively). The parents, the grand parents and other relatives of the three affected individuals had no homocystinuric symptoms, exhibiting a pattern of recessive inheritance in this family.

### Mutation screening of *CBS* in homocystinuria

**Figure 2:**
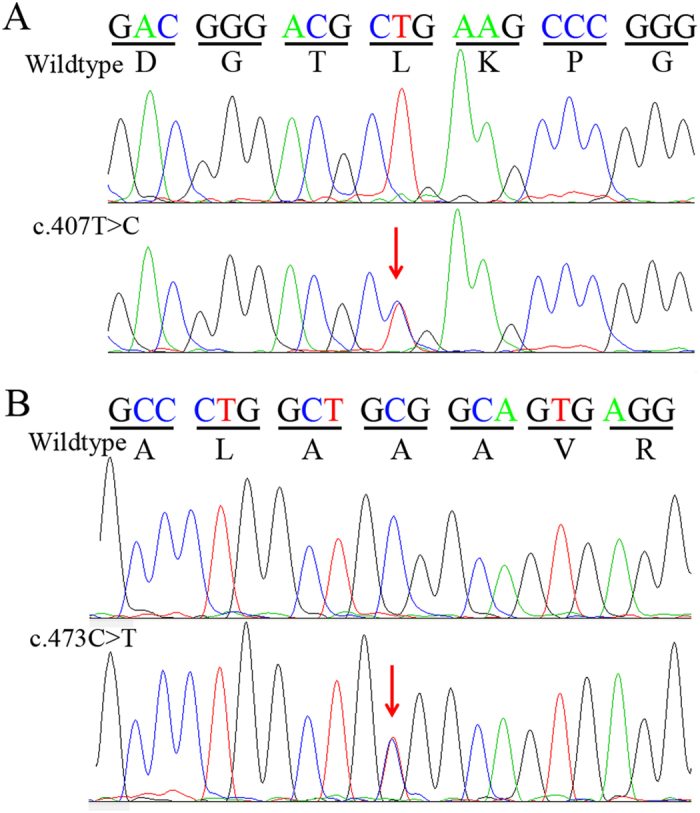
Direct sequencing results of *CBS* mutations. Direct sequencing identified two novel compound heterozygous mutations, (**A**) c.407T > C(p. L136P) and (**B**) c.473C > T (p.A158V)(indicated by red arrow).

**Figure 3:**
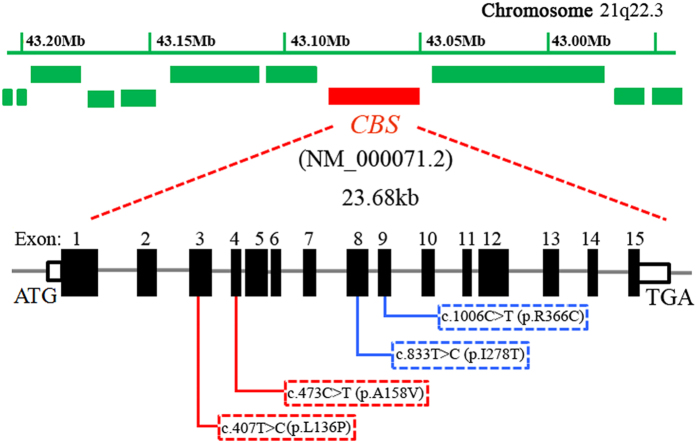
Mutations in the *CBS* gene identified in Chinese homocystinuric patients. The boxed mutations in red were newly found in this study and the mutations in blue box were identified in a Hong Kong homocystinuric patient.

Sequencing analysis of the *CBS* gene revealed novel compound heterozygous mutations, c.407T > C(p. L136P) and c.473C > T (p.A158V) (Fig. 2). They located in the coding sequence at nucleotide 407 of exon 3 and at nucleotide 473 of exon 4, respectively (Fig. 3). Both the two missense mutations were present in the three affected subjects (Table 3). The parents of the three patients were unaffected carriers with c.473C > T(father) and c.407T > C(mother) mutations, showing complete co-segregation of the mutations with the disease phenotype. Other available normal individuals in this family either carried one of the two mutations, or none. In addition, neither of the two missense heterozygous mutations was detected in 600 ethnically matched normal controls.

**Figure 4:**
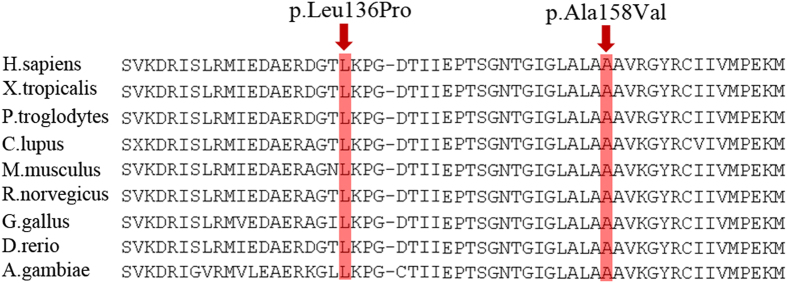
Orthologous protein sequence alignment of *CBS* from different species. The mutated residue showing conservation was shaded in red. Red shaded amino acids proteins showed that the two novel missense mutations occurred at highly conserved positions in these species.

Comparative amino acid sequence alignment of other *CBS* protein across different species revealed that the two novel mutations occurred at highly conserved positions (Fig. 4). Both of the two novel mutations could result in substitutions of amino acid in the *CBS* protein, and were predicted to be damaging by SIFT and Polyohen 2 (Table 3). The c.407T > C mutation is a T-C transition, converting Leucine (L) to Proline (P) at amino acid 136 (p. L136P); another mutation is a C-T transition (c.407T > C), leading to substitution of Alanine (A) toValine (V) at codon 158 (p.A158V, Fig. 2).

## Discussion

Homocystinuria is the most common inborn disorder of sulfur amino acid metabolism. *CBS* deficiency, a main factor causing homocystinuria, is an autosomal recessively inherited genetic defect. Since the first mutation in the human *CBS* gene reported by Kozich and Kraus in 1992^4^, many *CBS* mutations in homocystinuric patients from various populations worldwide have been identified. The present study identified novel compound heterozygous mutations, c.407T > C (p. L136P) and c.473C > T (p.A158V), in a Han Chinese family with homocystinuria and this result expands the spectrum of *CBS* mutations resulting in homocystinuria.

The human *CBS* gene, located at chromosome 21q22.3^16^, consists of 63-kDa subunits and encodes an enzyme with 551 amino acids^17^. The enzyme’s structure consists of a catalytic domain with 409 amino acids in the N-terminal and a regulatory domain with 142 amino acids in the C-terminal^18^. The protein encoded by this gene acts as a homotetramer to catalyze the conversion of homocysteine to cystathionine, the first step in the transsulfuration pathway^1^. The encoded protein is allosterically activated by adenosyl-methionine and uses pyridoxal phosphate as a cofactor. Defects in this gene can cause *CBS* deficiency, which can lead to homocystinuria. And most of affected patients are compound heterozygotes of these *CBS* mutations^10^,^14^,^15^,^19^. Until now, molecular genetic analyses of *CBS* deficiency have identified more than 150 pathogenic mutations among homocystinuric patients, mostly in the Caucasian populations and very few in African-Americans and Asians^20^. In 2011, two *CBS* mutations (c.833T > C and c.1006C > T) were detected in a Hong Kong homocystinuric patient by Kwok *et al.*^19^, however, it is so far the only report describing mutations in the *CBS* gene in Chinese (Fig. 2). In addition to the mutations identified in this study, the spectrum of mutations in *CBS* observed Han Chinese bears less resemblance to those observed in in Japanese and Korean patients^7^,^9^.

In this study, mutation analysis of three patients with homocystinuria in a Han Chinese family is described and we identified novel compound heterozygous for mutations c.407T > C (p. L136P) in exon 3and c.473C > T (p.A158V) in exon 4 of the *CBS* gene (Fig. 3). So far, these two mutations are reported in homocystinuric patients for the first time in mainland Han Chinese, although *CBS* mutations have been identified in different ethnic groups. In this pedigree, all the three affected patients (III:2, III:3 and III:4) were found to harbor both of the two missense mutations in *CBS*. The patients in this family were diagnosed as homocystinuria based on detection of elevated blood homocysteine, and diagnosis of skeletal deformities, mental retardation and ectopialentis. The proband of this family (III:3) presented with reduced vision, and were diagnosed by Provincial Hospital Affiliated to Shandong University at the age of 15. Biocularlens dislocation, high myopia, exotropia, glaucoma and corneal staphyloma were proved at that time. She had a history of bilateral downward dislocation of the lens since 7years old. No significant family history was noted except her two sisters (III:2 and III:4), who exhibited similar clinical manifestations with the proband. Surgeries were performed to extract the dislocated lens during 8 to 11 years old in these three affected girls. Their plasma total homocysteine level and methionine level were both markedly elevated, confirming the diagnosis of homocystinuria (Table 3). Molecular genetic testing of the *CBS* gene also helps to confirm the diagnosis of patients. Mutation analysis was also performed on the patient’s parents as well as her younger sister and brother, who are all unaffected (Fig. 1). Sequencing analysis showed that the father (II:3) and her sister (III:5)only carried thec.473C > T (p.A158V) mutation. Her mother (II:4) and brother(III:6) were heterozygous for thec.407T > C (p. L136P) mutation (Table 3).In addition, none of the two mutations in *CBS* was detected in 600 normal controls through gene analysis. Considering that *CBS* deficiency is an autosomal recessive disorder and that no other alteration was detected in coding regions of the *CBS* gene in homocystinuric patients of this family, it is highly possible that the two novel mutations of *CBS* identified here are responsible for the pathogenesis of homocystinuria in this pedigree.

For the p.L136P mutation identified in this pedigree, Leucine was replaced by Proline in exon 3, which is the most evolutionary conserved part of the *CBS* enzyme. Among all mutations identified in homocystinuric patients, about 25% mutations were in this conserved region (the third exon of this gene)^5^,^6^,^7^,^8^,^9^,^10^,^11^,^12^. The p.A158V mutation of *CBS* resulted in a substitution of Alanine to Valinein exon 4. Moreover, they are both predicted to be probably damaging to protein function by SIFT and PolyPhen-2. However, the exact mechanisms and pathological roles of the two novel mutations in *CBS* in the development of homocystinuria are largely unknown. In addition, the spectrum of mutations observed in this study bears less resemblance to those observed in Japanese^9^, Korean^7^ and Filipino^10^ patients, as well as Western countries, suggesting possible existence of ethnic differences among various populations. Future studies on Chinese homocystinuric subjects may help to provide more evidence for this hypothesis. In order to better understand homocystinuria pathogenesis, functional studies are needed to illustrate the role of *CBS* and the underlying mechanisms of this disease.

These data, together with the clinical presentation of the three affected siblings, demonstrated that p.L136Pandp.A158Vmutations in the *CBS* gene were responsible for homocystinuria in a Han Chinese family. Our data of *CBS* mutations causing homocystinuria further confirm the role of *CBS* in the pathogenesis of homocystinuria. This study expands the mutation spectrum of *CBS* resulting in homocystinuria, which could provide insights into the pre-symptomatic molecular diagnosis, the management of homocystinuric patients, and the genetic counseling of families in Chinese.

## Additional Information

**How to cite this article**: Gong, B. *et al.* Novel Compound Heterozygous *CBS* Mutations Cause Homocystinuria in a Han Chinese Family. *Sci. Rep.* **5**, 17947; doi: 10.1038/srep17947 (2015).

## Figures and Tables

**Table 1:** Clinical data of affected members in this family with homocystinuria.

Patient number	III:2	III:3	III:4
Age (Year)/Sex^1^	28/F	26/F	25/F
Onset age (Year)	5	7	5
Eye involvement	Biocular lens dislocation (were extracted 11 years ago); Myopia;Exotropia; Corneal staphyloma; Retinal detachment	Biocular lens dislocation (were extracted 11 years ago); Myopia; Exotropia; Corneal staphyloma	Biocular lens dislocation (were extracted 8 years ago); Myopia; Exotropia; Corneal staphyloma
Skeletal system	Kyphoscoliosis	Arachnodactylies	Arachnodactylies; Kyphoscoliosis; Mild pectuscarinatum
IQ^2^	Mental retardation; Dysarthria	Mental retardation; Dysarthria	Mental retardation; Dysarthria
Other signs	Pyramidal signs;Ataxia;Unstable gait;Brain atrophy; Malar flush	Pyramidal signs;Ataxia; Unstable gait;Brain atrophy; Malar flush	Pyramidal signs; Ataxia; Unstable gait;Brain atrophy; Malar flush
tHcy^3^	103	97	86
P-Met^4^	345	287	295

^1^F, female; M, male.

^2^IQ, intelligence quotient (assessed at presentation, various tests were used).

^3^tHcy, plasma total homocysteine level at presentation (μ mol/L), reference range 5–15 μ mol/L.

^4^P-Met, plasma methionine level at presentation (μ mol/L), reference range 20–40 μ mol/L.

**Table 2:** Primers used for mutation screening in *CBS* gene.

Primer Name	Primer Sequence(5′–3′)	Product Size(bp)	AnnealingTemperature (°C)
*CBS* 1F	CTCTCTCCTTGCTTTGCCAG	469	59
*CBS* 1R	CTGAGCATCCACTGTCTTGC		
*CBS* 2F	ATGTGTGTTTCAGGCGTGTG	453	59
*CBS* 2R	GCCACTCATTAACCAGCGAG		
*CBS* 3F	GGGGAGAAGCTCTGATAGGC	516	59
*CBS* 3R	CCGAATGCTGGTCAAAGGAA		
*CBS* 4&5&6F	CCATGTTGGGCAATTTTGGA	772	59
*CBS* 4&5&6R	AGCATTTCACAGAGGGAACA		
*CBS* 7F	CTTTCACAGACCAAGGGCAG	400	65
*CBS* 7R	TCTTCCCAAACACCTCCCAG		
*CBS* 8F	TTGGGTTTCTCATCCTGCCT	448	59
*CBS* 8R	GACCTTCGAGACCAGCTTCT		
*CBS* 9F	CTGTCTGCAAAACGTGTTGG	400	59
*CBS* 9R	CGCAGTGACACTCCTCAGAA		
*CBS* 10F	GCACAAGGAAGAAGCCGATG	366	59
*CBS* 10R	GTGAGAGGCATCCAGGGAAG		
*CBS* 11&12F	GCATGCTCACACACGCTT	819	59
*CBS* 11&12R	TGCCCTGAACGTCTGTATGA		
*CBS* 13F	CGAGGACATGTCTGACAGCA	975	65
*CBS* 13R	GAGTACTCTGGCACCCTCTG		
*CBS* 14F	CTGCCCAAACCTAGGAGTGA	432	59
*CBS* 14R	ACTGGGTGTCACTGAAGGTC		
*CBS* 15F	GGAGTCTGAGGCACGAGAAT	432	65
*CBS* 15R	GAAAGCGAAGGAGAAGTGGG		

F: Forward primer; R: Reverse primer; bp: Base pair.

**Table 3:** *CBS* mutations identified in this family with homocystinuria.

Mutation	Position^a^	Exon	NucleotideChange	Amino acidchange	SIFTscore	PolyPhenscore	Prediction^b^	Mutationtype^c^	MutaionStatus	Mutationpresented in^d^
L136P	44486397	3	c.407T > C	L136P	0	1.0	Damaging	Het	Novel	III:2, III:3, III:4, III:6, II:4, II:6, I:4
A158V	44485784	4	c.473C > T	A158V	0	1.0	Damaging	Het	Novel	IV:1, III:2, III:3, III:4, III:5, II:3, I:1

^a^Genomic positions are presented according to NCBI build 36.

^b^The SIFT and PolyPhenscore predict phenotypic effect.

^c^Het: heterozygous mutation.

^d^Subject number in this family with homocystinuria.
